# Complete Genome Sequence of *Bacillus* sp. Strain KH172YL63, Isolated from Deep-Sea Sediment

**DOI:** 10.1128/MRA.00291-20

**Published:** 2020-04-16

**Authors:** Yumi Murai, Takahiro Masuda, Yasuhide Onuma, Daniel Evans-Yamamoto, Nao Takeuchi, Hideto Mori, Nanami Masuyama, Soh Ishiguro, Nozomu Yachie, Kazuharu Arakawa

**Affiliations:** a Institute for Advanced Biosciences, Keio University, Tsuruoka, Japan; b Systems Biology Program, Graduate School of Media and Governance, Keio University, Fujisawa, Japan; c Synthetic Biology Division, Research Center for Advanced Science and Technology, The University of Tokyo, Tokyo, Japan; d Faculty of Environment and Information Studies, Keio University, Fujisawa, Japan; e Department of Biological Sciences, School of Science, The University of Tokyo, Tokyo, Japan; f PRESTO, Japan Science and Technology Agency (JST), Tokyo, Japan; g Exploratory Research Center on Life and Living Systems, National Institutes of Natural Sciences, Myodaiji, Okazaki, Japan; DOE Joint Genome Institute

## Abstract

*Bacillus* sp. strain KH172YL63 is a Gram-positive bacterium isolated from the deep-sea floor surface sediment at 3,308 m below sea level in the Nankai Trough in Japan. Here, we report the complete genome sequence of *Bacillus* sp. strain KH172YL63, which has a genome size of 4,251,700 bp and a G+C content of 44.8%.

## ANNOUNCEMENT

In the past few decades, there has been an increasing interest in marine organisms as possible sources of bioactive compounds, e.g., proteases (1) and alkaline cellulases (2). *Bacillus* species have been isolated from a huge variety of environments, both terrestrial and aquatic, including the model species Bacillus subtilis. *Bacillus* sp. strain KH172YL63 (phylum *Firmicutes*, class *Bacilli*, order *Bacillales*, family *Bacillaceae*) is a moderately psychrotrophic bacterium isolated from seawater of deep-sea sediment 3,308 m below sea level in the Nankai Trough in Japan (33°27′005ʺN, 137°16′990ʺE). Sediments from the sea bed were collected using a multiple corer, which collects sediments 0 to 1 m below the sea floor. We resuspended 100 mg of the dissolved mud samples in 3 ml of artificial seawater, plated dilution series onto modified seawater medium plates (360.75 mM NaCl, 7.5 mM KCl, 8.25 mM CaCl_2_ · 2H_2_O, 18 mM MgCl_2_ · 6H_2_O, 0.75 mM NaHCO_3_, 10.5 mM MgSO_4_ · 7H_2_O, 5.0% [wt/vol] Bacto peptone, 3.0% [wt/vol] yeast extract, and 1.5% Bacto agar), and incubated them at 20°C for single colony isolation. Colonies were obtained from samples collected 0 m below the sea floor.

*Bacillus* sp. KH172YL63 was inoculated onto a modified seawater medium (360.75 mM NaCl, 7.5 mM KCl, 8.25 mM CaCl_2_ · 2H_2_O, 18 mM MgCl_2_ · 6H_2_O, 0.75 mM NaHCO_3_, 10.5 mM MgSO_4_ · 7H_2_O, 5.0% [wt/vol] Bacto peptone, 3.0% [wt/vol] yeast extract, and 1.5% Bacto agar) plate for the isolation of single colonies. Taxonomic identification was performed using Sanger sequencing of amplified 16S rRNA genes, and a BLAST search matched with 99.93% identity to *Bacillus* sp. strain CNJ817 PL04. Genomic DNA was extracted using a Genomic-tip 20/G kit (Qiagen), and extracted DNA was used for both Nanopore and Illumina sequencing. The long reads for *Bacillus* sp. KH172YL63 were generated with GridION sequencing (Oxford Nanopore Technologies), where the sequencing library was prepared using the rapid barcoding kit (SQK-RBK004) and was run in a FLO-MIN106 flow cell. For Illumina sequencing, a library of fragmented genomic DNA was prepared using a HyperPlus library preparation kit (KAPA Biosystems) and sequenced on a NextSeq 500 sequencer with high-output mode and 75 cycles (Illumina). A total of 251,000 (*N*_50_ value, 19 kbp; maximum length, 183 kbp) and 79.7 million reads were obtained for the Nanopore and Illumina sequencing, respectively.

The genome sequence was obtained by *de novo* assembly using raw Nanopore reads with default settings of the Canu assembler v1.8.0 (3). An improved consensus sequence for the draft assembly was obtained with raw Illumina reads and the Pilon software v1.23 using default parameters (4). The assembly resulted in a single contig spanning the entire chromosome, with a total length of 4,251,700 bp and a G+C content of 44.8%. Automatic genome annotation was performed with DFAST (DDBJ Fast Annotation and Submission Tool) (5). We identified 4,243 coding sequences (CDSs), 108 tRNAs, and 33 rRNAs. The assembly completeness was assessed with BUSCO v1 (6) on the gVolante server (7), resulting in 100% completeness.

*Bacillus* sp. KH172YL63 has several proteases, such as germination protease, an ATP-dependent Clp protease, and the ATP-binding subunits ClpE and ClpX. These proteases may be applied in industrial processes (8). The complete genome sequence of *Bacillus* sp. KH172YL63 reported here may facilitate proteases studies.

### Data availability.

The complete genome sequence of *Bacillus* sp. KH172YL63 has been deposited in DDBJ/GenBank under the accession number AP022842. The assembly and raw reads are collected together under BioProject number PRJNA606022.
